# Autism spectrum disorder in Phelan-McDermid syndrome: initial characterization and genotype-phenotype correlations

**DOI:** 10.1186/s13023-015-0323-9

**Published:** 2015-08-27

**Authors:** Lindsay M. Oberman, Luigi Boccuto, Lauren Cascio, Sara Sarasua, Walter E. Kaufmann

**Affiliations:** Department of Neurology, Boston Children’s Hospital, and Harvard Medical School, 300 Longwood Avenue, Boston, MA 02115 USA; Neuroplasticity and Autism Spectrum Disorder Program, E.P. Bradley Hospital and Warren Alpert Medical School of Brown University, 1011 Veterans Memorial Parkway, East Providence, RI 02915 USA; JC Self Research Institute, Greenwood Genetic Center, 113 Gregor Mendel Circle, Greenwood, SC 29646 USA

**Keywords:** Phelan-McDermid Syndrome, Autism Spectrum Disorder, SHANK3, 22q13

## Abstract

**Background:**

Phelan-McDermid syndrome (PMS) is a neurodevelopmental disorder associated with a terminal deletion affecting chromosome 22 (22q13) that results in the loss of function of the SHANK3 gene. SHANK3 has also been identified in gene-linkage studies to be associated with autism spectrum disorder (ASD). Diagnosis of ASD in individuals with PMS is complicated by the presence of moderate to profound global developmental delay/intellectual disability as well as other co-morbid systemic and neurological symptoms.

**Methods:**

The current study aimed to characterize the symptoms of ASD in patients with PMS and to do a preliminary exploration of genotype-ASD phenotype correlations. We conducted a standardized interview with 40 parents/guardians of children with PMS. Further, we conducted analyses on the relationship between disruption of *SHANK3* and adjacent genes on specific characteristic symptoms of ASD in PMS in small subset of the sample.

**Results:**

The majority of PMS participants in our sample displayed persistent deficits in Social communication, but only half met diagnostic criteria under the restricted, repetitive patterns of behavior, interests, or activities domain. Furthermore, logistic regressions indicated that general developmental delay significantly contributed to the ASD diagnosis. The analyses relating the PMS genotype to the behavioral phenotype revealed additional complex relationships with contributions of genes in both deleted and preserved *SHANK3* regions to the ASD phenotype and other neurobehavioral impairments.

**Conclusions:**

There appears to be a unique behavioral phenotype associated with ASD in individuals with PMS. There also appears to be contributions of genes in both deleted and preserved *SHANK3* regions to the ASD phenotype and other neurobehavioral impairments. Better characterization of the behavioral phenotype using additional standardized assessments and further analyses exploring the relationship between the PMS genotype and behavioral phenotype in a larger sample are warranted.

**Electronic supplementary material:**

The online version of this article (doi:10.1186/s13023-015-0323-9) contains supplementary material, which is available to authorized users.

## Background

Autism Spectrum Disorder (ASD) is a behaviorally defined complex neurodevelopmental disorder. According to the Diagnostic and Statistical Manual of Mental Disorders (DSM-5), the core symptoms include qualitative impairments in social communication and the presence of restricted and repetitive behaviors, interests and activities. Though the exact etiology is unknown, recent studies have identified over 100 genes and recurrent genomic imbalances implicated in the etiology of ASD [1]. Though it seems that the genetic contribution to ASD is complex, accumulating evidence suggests that the risk genes ultimately converge on a relatively small set of molecular pathways, including those critical for synaptic development and plasticity [2]. One such risk gene is the SH3 and multiple ankyrin repeat domains 3 (*SHANK3*) gene [3–5], which lies on the distal long arm of chromosome 22 and whose protein product (by the same name) acts as a scaffolding protein in its interactions with various synaptic molecules, including the N-methyl-D-aspartate receptor (NMDA-R), class I metabotropic glutamate receptors (mGluRs), and the GluR1 a-amino-3-hydroxy-5-methyl–4-isoxazole propionic acid receptor (AMPA-R) [6]. A recent study reported that a screen for *SHANK3* mutations in 3,833 individuals with ASD and Intellectual disability (ID) revealed a prevalence of a mutation in the *SHANK3* gene in over 2 % of this group [7]. Estimates of the prevalence of ASD in patients with known abnormalities in the region of the *SHANK3* gene (i.e., patients with 22q13 deletion syndrome or Phelan-McDermid Syndrome (PMS)) are conflicting with published studies reporting anywhere from 0–94 % depending on how the information was obtained [8–10].

Clinical reports of patients with PMS highlight a wide range of systemic and neurologic manifestations, the latter including moderate to profound global developmental delay/intellectual disability, hypotonia, absent or delayed speech, seizures, and decreased perception of pain [8, 11, 12]. Physical manifestations include minor dysmorphic features, increased incidence of lymphedema, cardiac abnormalities, and renal abnormalities [8, 11]. Related to the prevalence of ASD in this population, their behavior is described as impaired communication and impaired social interactions with the presence of self-stimulatory behavior [8].

The aim of this initial study was to systematically characterize the neurobehavioral profile as it relates to symptoms of ASD in patients with PMS and to explore genotype-ASD phenotype correlations. We conducted a standardized interview with 40 parents/guardians of children with PMS ages 3–18 that included the Autism Diagnostic Interview-Revised (ADI-R) [13] and the Vineland Adaptive Behavior Scale Second Edition (Vineland II) [14]. Further, in a subgroup of patients, for whom we had access to DNA data, we were able to conduct analyses on the relationship between disruption of *SHANK3* and adjacent genes on specific characteristic symptoms of ASD in PMS. In addition to examining the relationship between *SHANK3* region deletion, we evaluated the potential contribution of the non-deleted copies of 22q13 genes that might carry sequence variants modulating SHANK3 protein’s function. Although *SHANK3* haploinsufficiency, or epigenetic dysregulation of the gene, appears to play a major role in determining the ASD phenotype in PMS, it has been shown that other regions of 22q13 may play a role in the neurobehavioral phenotype of the syndrome [12, 15, 16]. Thus, it is unclear whether *SHANK3*’s loss of function is sufficient for determining ASD or other genes in the region may also play a primary or modifying role. Several of the genes in the *SHANK3* region are appealing candidates because of their demonstrated function: *IB2* encodes a scaffold protein enriched in postsynaptic densities and regulates glutamatergic transmission in the cerebellum [17], some genes have brain-specific expression patterns (*IB2*, *RABL2B*), or roles in *SHANK3* regulation (micro-RNA *has-mir124910*)*.* Based on the previous literature, we hypothesized that the children with PMS would show moderate-profound global developmental delay or intellectual disability and symptoms consistent with the DSM-5 criteria of ASD. It was unclear which symptoms would be more severe or what percent of the sample would meet all of the diagnostic criteria for ASD. Additionally, DSM-5 criteria for ASD requires that that the social communication deficit and other diagnostic impairments must be above what may be expected based on the individual’s intellectual or communication abilities [18, 19]. Thus, in this population, and others where there is a global developmental delay, it is critical that ASD diagnosis be made in the context of the individual’s general intellectual or communicative skills. Previous studies in our laboratory have similarly aimed to characterize the neurobehavioral profile of ASD in the context of other genetic disorders that often result in both ASD symptoms as well as global developmental delay, namely fragile X syndrome and Down syndrome [20, 21]. Interestingly, previous studies had indicated that patients with PMS that meet criteria for ASD appear to have smaller deletions restricted only to the *SHANK3* region, while those who did not meet criteria and have a more severe phenotype had larger deletions that also affected other genes [8, 16]. This may be a result of smaller deletions resulting in less severe global delays and thus more clear demonstration of specific deficits in the social communication domain. Additionally, no data are available about the role of the non-deleted *SHANK3* region genes. Thus, the relationship between *SHANK3* deficit and ASD in PMS appears to be quite complex requiring careful evaluation using standardized, well-validated measures.

## Methods

### Participants

We recruited a cohort of 40 parents/guardians of children with PMS in the 3–18 years age range (M = 9.95 years, SD = 4.46 years; 25 Males, 15 Females). Recruitment was assisted by the Phelan-McDermid Syndrome Foundation. A registry of patients with PMS (with a 22q deletion or *SHANK3* mutation) is maintained by the Foundation. Diagnosis was based on clinical reports supplied by the parents. Out of the total sample of 40 children: Thirty-one had deletions affecting the 22q13 region of chromosome 22; Two had complex chromosomal rearrangements including a deletion in 22q13 region of chromosome 22; Three had 22ring chromosomes; One had an unbalanced translocation involving chromosomes 22 and 18; And One had a point mutation in 22q13 region of chromosome 22. Participants self-selected themselves to be part of the study, based on an announcement that was sent out to all members of the registry. Enrollment was not biased toward whether their diagnosis was a result of a point mutation or deletion, or the size or exact location of their mutation or deletion, or whether they showed symptoms of ASD. This study was reviewed and approved by the Boston Children’s Hospital Internal Review board, the Committee on Clinical Investigation.

### Procedure

The study involved a single phone interview with at least one parent/guardian of the affected individual. The interview consisted of the ADI-R and the Vineland II, which was administered by a trained psychologist. The ADI-R is a standardized interview commonly used for diagnosing autism, planning treatment, and distinguishing autism from other developmental disorders [13]. It is composed of 93 items, focusing on three functional domains: 1. Language/Communication; 2. Reciprocal Social Interactions; and 3. Restricted, Repetitive, and Stereotyped Behaviors and Interests. Interview questions cover eight content areas: 1) The subject’s background, including family, education, previous diagnoses, and medications, 2) Overview of the subject’s behavior, 3) Early development and developmental milestones, 4) Language acquisition and loss of language or other skills, 5) Current functioning in regard to language and communication, 6) Social development and play, 7) Interests and behaviors, and 8) Clinically relevant behaviors, such as aggression, self-injury, and possible epileptic features. We re-structured the ADI-R items and subdomains to align with the new DSM-5 diagnostic criteria for ASD; one of the authors (WEK) was a member of DSM-5’s Neurodevelopmental Disorders Work Group that drafted the revised criteria. The Vineland II is a leading instrument for supporting the diagnosis of intellectual disability, particularly after the DSM-5 revision [14, 19]. The scales of the Vineland II are organized within a three-domain structure: Communication, Daily Living, and Socialization. This structure corresponds to the three broad domains of adaptive functioning by the American Association of Intellectual and Developmental Disabilities: Conceptual, Practical, and Social. In addition, Vineland II offers a Motor Skills Domain and an optional Maladaptive Behavior Index to provide more in-depth information. Both the ADI-R and Vineland II have good reliability and validity when administered over the phone [22, 23].

For the 14 patients for whom DNA samples were available at the Greenwood Genetics Center from a previous study, we were able to amplify by PCR and screen by Sanger sequencing the coding regions (including intron/exon boundaries) of the following genes: *SHANK3* (22 exons), *MAPK8IP2*/*IB2* (13 exons), *RABL2B* (8 exons), *hsa-miR-1249* (1 exon). The sequences of the oligonucleotide primers used to sequence the abovementioned genes are reported in the Additional file 1. The significance of the findings was evaluated by interrogation of bioinformatic websites and comparison with genomic variant databases (see Additional file 1 for websites addresses).

### Data analysis

Descriptive and Inferential statistics were computed based on modified subdomains of the ADI-R based on DSM-5 criteria and standardized Vineland II scores using SPSS version 22. The ADI-R was developed to match the DSM-IV criteria of autism with subdomains related to A) Qualitative Abnormalities in Reciprocal Social Interaction (including A1: Failure to use nonverbal behaviors to regulate social interaction, A2: Failure to develop peer relationships, A3: Lack of shared enjoyment, and A4: Lack of socioemotional reciprocity), B) Qualitative Abnormalities in Communication (including B1: Lack of or delay in spoken language and failure to compensate through gesture, B2 (only scored for verbal participants): Relative failure to initiate or sustain conversational interchange, B3 (only scored for verbal participants): Stereotyped, repetitive or idiosyncratic speech, and B4: Lack of varied spontaneous make-believe or social imitative play) and C) Restricted, Repetitive, and Stereotyped Patterns of Behavior (including C1: Encompassing preoccupation or circumscribed pattern of interest, C2: Apparently compulsive adherence to nonfunctional routines or rituals, C3: Stereotyped and repetitive motor mannerisms, and C4: Preoccupation with parts of objects or nonfunctional elements of material).

In order to fit with current DSM-5 criteria, the subdomains were reclassified as A) Persistent deficits in social communication and social interaction across multiple contexts including A1: Deficits in social-emotional reciprocity, which included the domains previously categorized under A4 (and B2 for verbal participants), A2: Deficits in nonverbal communicative behaviors used for social interaction, which included the domains previously categorized under A1 and B1, A3: Deficits in developing, maintaining, and understanding relationships, which included the domains previously categorized under A2 and B4, B) Restricted, repetitive patterns of behavior, interests, or activities (including B1: Stereotyped or repetitive motor movements, use of objects, or speech, which included domains previously categorized under C3 (and B3 for verbal participants), B2: Insistence on sameness, inflexible adherence to routines, or ritualized patterns or verbal nonverbal behavior, which matched to C2 in the ADI-R, B3: Highly restricted, fixated interests that are abnormal in intensity or focus, which matched to C1 in the ADI-R, and B4: Hyper- or hyporeactivity to sensory input or unusual interests in sensory aspects of the environment, which matched to C4 in the ADI-R. Averages per question scores were calculated to evaluate the degree of impairment in each of these reclassified domains. On the ADI-R each question is scored on a three-point scale with 0 indicating no impairment, 1 indicating some impairment, and 2 indicating significant impairment related to that specific behavior.

Logistic regression analyses were conducted on factors that contribute to the presence or absence of an ASD diagnosis, as it has been done previously in our laboratory to characterize the neurobehavioral profile of ASD in the context of other genetic disorders including fragile X syndrome and Down syndrome [20, 21]. Descriptive analyses as well as Pearson product mean correlation coefficients were calculated on the genetic analyses as well as the relationships between the genetic analyses and the behavioral phenotype.

## Results

### Behavioral results

**Fig. 1:**
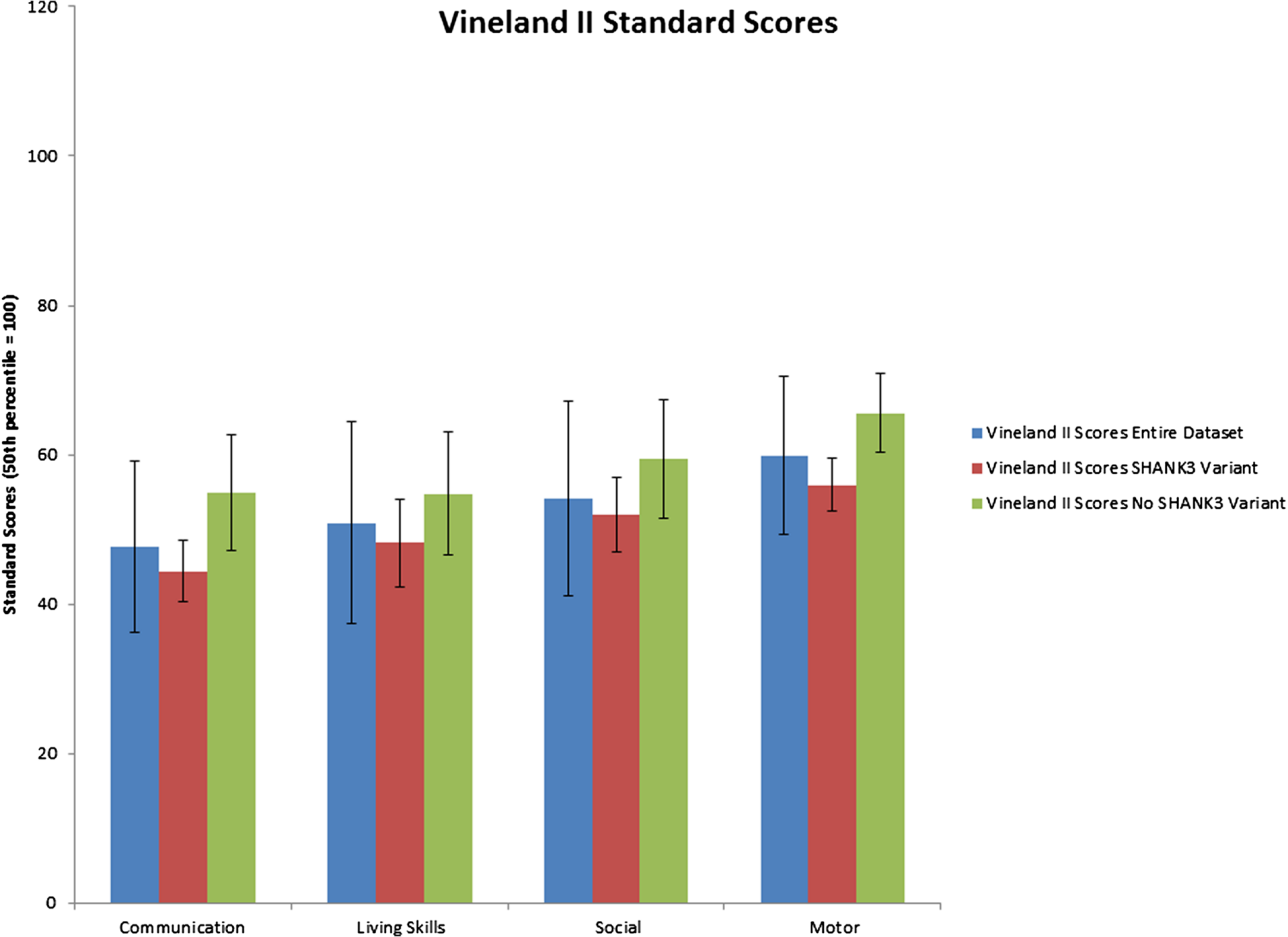
Standard scores for the Vineland II Communication, Living Skills, Social, and Motor domains. 50^th^ percentile in the general population is 100. Average Scores reflect mild to moderate impairments in adaptive skills in patients with PMS. Blue Bars represent the entire dataset, red bars represent the subgroup who we identified to carry *SHANK3* Variants green bars represent the subgroup who were confirmed *not* to carry a *SHANK3* Variant. Error Bars represent the Standard Error of the Mean

Group Average Vineland II standard scores (age norm 100) ranged from 48 (Communication subdomain score) to 60 (Motor subdomain score) with only one individual scoring in the average range (Fig. 1). None of the individuals appear to have spared or splinter skills, which together with the Vineland II scores suggest a global cognitive deficit (i.e., global developmental delay or intellectual disability). Most individuals (75 %) were nonverbal and 21 subjects (53 %) had a clinical diagnosis of ASD. Based on the ADI-R, the overwhelming majority of the sample (90 %) displayed persistent deficits in Social communication, the first DSM-5 diagnostic criteria for ASD.However, only approximately half of the sample (55 %) met diagnostic criteria for Restricted, repetitive patterns of behavior, interests, or activities (presence of symptoms in two domains), the second DSM-5 diagnostic criteria for ASD. Commonly seen in this sample were hyper- or hyporeactivity to sensory input or unusual interests in sensory aspects of the environment (DSM5-B4). This manifested as chewing behavior in 68 % of the sample and is consistent with previous reports of individuals with PMS [11]. A large proportion of the sample also had blunted facial expression, present in 73 % of participants. PMS subjects’ emotional facial expressions appear to be limited with a bias toward a happy expression. A history of epilepsy was present in 20 % of the sample. Loss of social communication skills was only observed in a single participant with no relationship with onset of seizures or other medical conditions, suggesting that regression is not common in the study population (Table 1).

**Table 1:** Sample characteristics (*N* = 40)

Sample characteristic	N	Percentage
Gender (Male:Female)	25:15	62.5 %:37.5 %
Clinical ASD Dx (Parent Report)	21	52.5 %
Language (Verbal: Nonverbal) (ADI-R)	10:30	25 %:75 %
Social Communication Deficit (ADI-R)	36	90 %
Restricted and Repetitive Behavior, Interests and Activities (ADI-R)	22	55 %
Sensory Seeking Activities (ADI-R)	27	68 %
Epilepsy (Parent Report)	8	20 %
Blunted Facial Expression (ADI-R)	29	73 %
True Regression (ADI-R)	1	2.5 %
Borderline Adaptive Behavior Delay (Vineland II)	2	5 %
Mild Adaptive Behavior Delay (Vineland II)	21	52.5 %
Moderate Adaptive Behavior Delay (Vineland II)	17	42.5 %

**Fig. 2:**
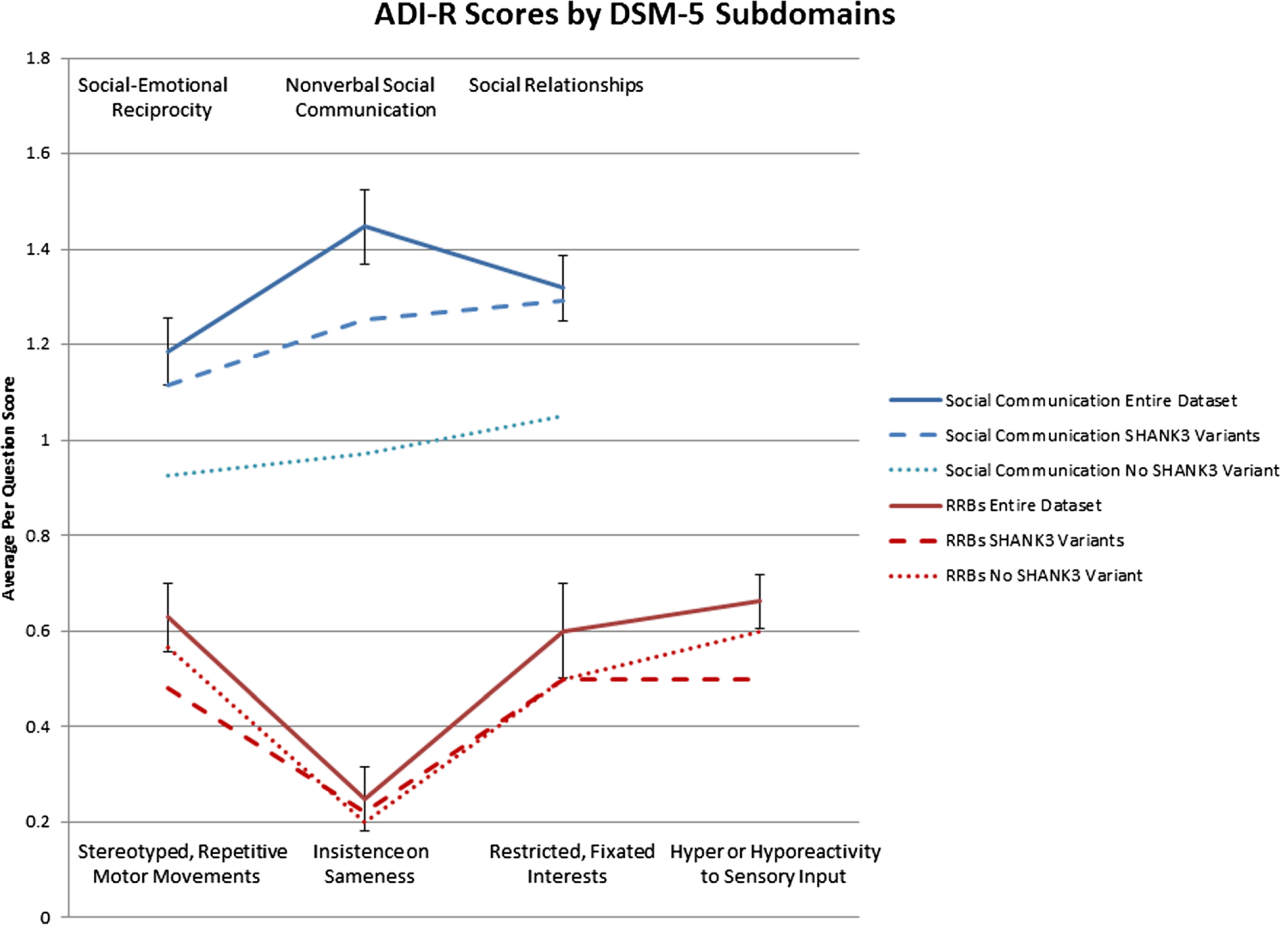
Average per question scores on the ADI-R subdomains that index the core symptoms of ASD based on the DSM5 criteria. A score of zero indicates no evidence of symptoms in that subdomain, a score of one indicates some evidence of symptoms in that subdomain, a score of two indicates definite evidence of symptoms in that subdomain. Patients in this sample display evidence of symptoms in the Social Communication domain, but relatively fewer symptoms in the Restricted and Repetitive Behavior (RRB) domain. Dashed lines indicate the mean values for the subgroup who we identified to carry *SHANK3* Variants. Dotted lines indicate the mean values for the subgroup who were confirmed *not* to carry a *SHANK3* Variant. Error Bars represent the Standard Error of the Mean

For all participants, an analysis of variance (ANOVA) was conducted on the seven subdomains of diagnostic criteria: A) Persistent deficits in social communication and social interaction across multiple contexts (including A1: Deficits in social-emotional reciprocity, A2: Deficits in nonverbal communicative behaviors used for social interaction, A3: Deficits in developing, maintaining, and understanding relationships), B) Restricted, repetitive patterns of behavior, interests, or activities (including B1: Stereotyped or repetitive motor movements, use of objects, or speech, B2: Insistence on sameness, inflexible adherence to routines, or ritualized patterns or verbal nonverbal behavior, B3: Highly restricted, fixated interests that are abnormal in intensity or focus, and B4: Hyper- or hyporeactivity to sensory input or unusual interests in sensory aspects of the environment). The ANOVA revealed a significant difference in degree of impairment across subdomains (F (6,234) = 52.244, *p* < 0.001) with the greatest impairment shown in DSM-5 Social communication and specifically Nonverbal communication used for social interaction (A2) (M = 1.45, SD = 0.49) and the least impairment shown in DSM-5 Restricted, repetitive behavior, interests or activities and specifically Insistence on sameness, inflexible adherence to routines, or ritualized patterns or verbal nonverbal behavior (B2) (M = 0.25, SD = 0.41) (Fig. 2).

A forward stepwise linear regression analysis was conducted in order to determine whether the persistent deficits in social communication could be accounted for by the overall developmental delay/intellectual impairment indicated by the Vineland II subdomain scores. The only score that significantly predicted the ADI-R Social communication domain score was the Vineland II Social subdomain score (Adjusted R^2^ = 0.39, β = −0.635, *p* < 0.001). A forward stepwise logistic regression analysis was conducted to determine which ADI-R or Vineland II scores significantly contributed to the presence or absence of a clinical ASD diagnosis. A test of the full model versus a model with the intercept only was statistically significant, *χ*^2^ (6, N = 40) = 14.13, *p* < 0.05 Negelkerke R^2^ = 0.397. The model was able to correctly classify 74 % of those who do not have ASD and 86 % of those who do, for an overall success rate of 80 %. Employing a 0.05 threshold for statistical significance, only the Vineland II Social subdomain score significantly contributed to the model (*b* = −0.236, Wald *χ*^2^ (1) = 7.123, *p* < 0.01).

### Genotype-phenotype relationships

Of the 40 individuals in the study, DNA samples were available for 14 (Mean Age = 11.69, SD 4.23). The average size of the deletion in these individuals was 5.01 MB (SD = 2.45) (range = 0.22–9.18). This subgroup had a similar behavioral profile as the larger group with 8/14 (57 %) having an clinical diagnosis of ASD and 64 % who were nonverbal. The ADI-R scores reflected persistent deficits in Social communication in 79 % and presence of Restricted, repetitive patterns of behavior, interests, or activities in two domains in 43 % of individuals in this subgroup. The Vineland II scores also matched the larger group with average subdomain standard scores ranging from 48 (Communication subdomain score) to 59 (Motor subdomain score).

Pearson Correlation Coefficients were calculated to evaluate the association between size of deletion and behavioral phenotype. Significant associations and trends were observed for both the ADI-R and Vineland II scores: ADI-R DSM-5 Restricted, repetitive patterns of behavior, interests, or activities was negatively correlated with size of deletion (r(14) = −0.48, *p* = 0.09); Vineland II Communication subscore (r(14) = −0.57, *p* = 0.03); Vineland II Motor subscore (r(14) = −0.68, *p* < 0.01) and Vineland II Living Skills subscore (r(14) = −0.51, *p* = 0.06) were also negatively correlated. Note that all relationships were negative, indicating more severe restricted and repetitive behavior was associated with a smaller deletion size but larger genetic loss with more impaired general adaptive behavior skills. Also note that neither the Social Communication subscore on the ADI-R nor the Social subdomain on the Vineland II significantly correlated with deletion size.

A second set of analyses, illustrated in Table 2, examined the allelic non-deleted *SHANK3* region for a possible “second hit” phenomenon. Nine out of fourteen individuals displayed variants that have been previously associated with ASD. Eight out of fourteen subjects (57 %) carried the *SHANK3* c.1612 + 18C > T substitution in intron 13, which is predicted to cause abnormal splicing. One additional participant carried the *SHANK3* c.1304 + 48C > T substitution in intron 10, which removes a CpG island in one of the four intragenic regulatory elements and has been reported to be associated with ASD [5]. The group with the *SHANK3* variants had more severe social communication deficits and more impaired adaptive skills, as compared to the remaining six patients not carrying the change, though these differences failed to reach significance. Analysis of other genes in the *SHANK3* region was restricted to profiling one subject with the *IB2* c.595G > A substitution in intron 13, not previously described in any variant database. This individual also carried the *SHANK3* c.1612 + 18C > T substitution and displayed significantly less severe social communication deficits on the ADI-R, but significantly more severe impairments in adaptive behavior skills on the Vineland II (all ps <0.01).

**Table 2:** SHANK3 and IB2 variants in PMS patients (*n* = 14)

Sample	Size of 22q13 terminal deletion (Mb)	Variant	Effect	Per question average score in the social communication domain (ADI-R)	Per question average score in the restricted and repetitive behavior, interests and activities domain (ADI-R)	Average Vineland II standard score	Notes
cms 20176a, cms16938, cms16941, cms13591, cms13583, cms13569, cms14279, cms13562	3.33035, 3.222768^a^,4.493653, 4.807163, 8.65534, 5.227017, 7.031477, 6.496731	*SHANK3* c.1612 + 18C > T (Intron 13)	Possible splice effect (reduced splice acceptor site affinity)	M = 1.18, (SD = 0.45)	M = 0.47 (SD = 0.27)	M = 52.3 (SD = 11.05)	rs1557620 C: 81 % T: 19 % (TT: 5.9 %) 1000 genomesC: 80.5 % T: 19.5 % (TT: 4.1 %) NHLBI
cms13661	9.183577	*SHANK3* c.1304 + 48C > T (Intron 10)	Lost 1 CpG	1.62	0.25	33	rs76224556 C: 98 %, T: 2 % (TT: 0.1 %) 1000 genomes C: 98.3 %, T: 1.7 % (TT: 0.04 %) NHLBI
cms13583	8.65534	*IB2* c.594G > A (Exon 5) and *SHANK3* c.1612 + 18C > T (Intron 13)	p.Ser198Ser and Possible splice effect (reduced splice acceptor site affinity)	0.62	0.5	40	Novel
cms13465, cms13436, cms13459, cms24824, cms20670	4.848708, 2.79066, 3.088414, 6.718459, 0.219087	None	N/A	M = 0.99 (SD = 0.51)	M = 0.52 (SD = 0.47)	M = 58.7 (SD = 13.6)	N/A

^a^Patient also has 0.652424 Mb duplication in 22q13 region

## Discussion

The goal of the current study was to perform an initial characterization of the ASD behavioral phenotype, and its genotypical correlations, in individuals with PMS. The results indicate that the subjects in our sample had a complex behavioral phenotype, most notably characterized as mild to moderate generalized developmental delay/intellectual disability. As it relates to autism specific symptoms, the majority of the individuals displayed persistent deficits in Social communication, but only half met diagnostic criteria under the Restricted, repetitive patterns of behavior, interests, or activities domain. In the context of the general delay/deficit in adaptive behavior shown in this population, it is imperative to recognize that the DSM-5 is more explicit in that the social communication deficit and other diagnostic impairments must be above what may be expected based on the individual’s intellectual or communication abilities [18, 19]. This is especially critical as logistic regressions indicated that the main factor that contributed to the clinical diagnosis of ASD was the Social subdomain from the Vineland II, rather than any factor from the ADI-R. Thus, additional testing will need to be conducted in order to evaluate what proportion, for instance, of the social communication deficits are accounted for by the general developmental delay in each individual.

Some suggested measures would include a standardized IQ assessment such as the Stanford-Binet, Fifth Edition (SB5). Other standardized measures such as the Peabody Picture Vocabulary Test and the Expressive Vocabulary Test could be used to quantify the degree of impairment in receptive and expressive language skills. The Autism Diagnostic Observation Schedule, Second Edition (ADOS-2) combined with the ADI-R is considered the gold-standard for ASD diagnosis, thus, when feasible, ASD diagnosis should incorporate the ADOS-2. Another standardized assessment found to identify salient non-diagnostic, but associated features of ASD is the Aberrant Behavior Checklist (ABC). The ABC Irritability subscale would be specifically interesting in light of the observation in this sample that despite having significant social communication deficits including limited emotional facial expression, participants generally had a happy demeanor.

The analyses relating the PMS genotype to the behavioral phenotype revealed additional complex relationships. Consistent with other reports, there appeared to be a trend toward a negative relationship between size of deletions and the presence of repetitive and restricted patterns of behavior, interests and activities with smaller deletions leading to more severe phenotype [8, 16] while deletion size was not significantly associated with Social Communication deficits. These data suggest that specific gene deficits but not total loss of genetic material may explain ASD in PMS. An inverse correlation was also found between deletion size and adaptive communication, motor and living skills indicating that, nonetheless, magnitude of gene loss matters since larger deletions were associated with greater adaptive skill impairment. Considering that our cohort had marked impairment in adaptive communication (Table 1) and that these skills are highly correlated with overall cognitive function in autistic and non-autistic individuals [24, 25], our data suggest that general developmental delay may have masked the contribution of other genes in the deleted *SHANK3* region to ASD symptoms. A novel aspect of the present study is the examination of the role of the preserved *SHANK3* region in the PMS phenotype. For the first time, the hypothesis of a “second hit” model involving 22q13 genes has been tested. The selected genes have brain-specific expression and are involved in synaptic function and neuronal development. The rationale behind our analysis was based on the possibility that sequence variants, even if not clearly deleterious, could affect the function of the preserved copy of those genes, modulating the neurobehavioral phenotype of the subject and contributing to the heterogeneity in the clinical presentation of PMS. Although preliminary, the association of the *SHANK3* c.1304 + 48C > T and *SHANK3* c.1612 + 18C > T variants with greater deficits in social communication and adaptive skills compared to those without these variants support a “second hit” phenomenon. Moreover, the observation of one subject with both the *IB2* c.595G > A variant and *SHANK3* c.1612 + 18C > T variant with milder social communication impairments, but more severe adaptive behavior skills, emphasizes the need for directly examining other genes in the *SHANK3* region in both deleted and preserved chromosome 22. Overall, 9 out of 14 subjects (64.3 %) presented at least one variant with potential effects on the protein function (Table 2), suggesting that 22q13 deletions may in some cases unmask rare autosomal recessive gene deficits and that the genetic contribution to the PMS phenotypical heterogeneity should be investigated beyond the mere haploinsufficiency.

The present study should be considered as preliminary in nature, providing directions for future investigations of the ASD phenotype and its genetic bases in PMS. Among its most important contributions are the use of standardized behavioral measures and the DSM-5 diagnostic approach to ASD. To our knowledge, the subject sample is the largest reported in behavioral studies of PMS. Nevertheless, the behavioral characterization was limited by relying on only two instruments and exclusively on interviews. Direct assessment and use of a comprehensive neurobehavioral battery described above are essential follow up steps, which we intend to pursue with the same cohort. The second highlight of this study is the analysis of genetic contributions beyond deletion size. The inclusion of the non-deleted *SHANK3* region was a revealing and promising line of inquiry. The genotype-phenotype correlations here underscore the complexity of examining the role of deleted genetic material and the need of having a more complete picture of the neurobehavioral phenotype. For instance, we could not rule out that magnitude of genetic loss does indeed have an impact on ASD severity because of the co-existence of severe adaptive behavior impairment. The relatively small subset of subjects with genotype data was an important limitation considering that our findings suggest the need for complex statistical models, which incorporate genes in both deleted and preserved *SHANK3* regions. Our future correlative studies will address the shortcomings discussed here.

## Conclusions

In summary, there appears to be a unique behavioral phenotype associated with ASD in individuals with PMS that warrants further investigation. Careful neuropsychological evaluations are necessary to ensure that the social communication deficits present in these individuals cannot be explained by their overall developmental delay/intellectual disability. These additional standardized measures assessing social communication and intellectual functioning will further validate the diagnosis of ASD in individuals with PMS. There also appear to be contributions of genes in both deleted and preserved *SHANK3* regions to the ASD phenotype and other neurobehavioral impairments, which need a more detailed examination with a larger sample.

## Additional file

Additional file 1:
**Oligonucleotide primers list.** (DOC 125 kb)

## Abbreviations

PMS: Phelan McDermid Syndrome
SHANK3: SH3 and multiple ankyrin repeat domains 3
ASD: Autism Spectrum Disorder
DSM-5: Diagnostic and Statistical Manual-Fifth Edition
NMDA-R: N-methyl-D-aspartate receptor
mGluRs: Metabotropic glutamate receptors
AMPA-R: a-amino-3-hydroxy-5-methyl-4-isoxazole propionic acid receptor
ADI-R: Autism diagnostic interview-revised
Vineland-II: Vineland Adaptive Behavior Scale-Second Edition
PCR: Polymerase chain reaction
CpG: Cytosine phosphate guanine
