# Supplementary material for: Autism spectrum disorder in Phelan-McDermid syndrome: initial characterization and genotype-phenotype correlations
Source: Orphanet J Rare Dis. 2015 Aug 27;10:105. doi: 10.1186/s13023-015-0323-9 (PMC4549933; doi:10.1186/s13023-015-0323-9)
Supplement: Additional file 1: — Oligonucleotide primers list. (DOC 125 kb) [file 13023_2015_323_MOESM1_ESM.doc]

**Supplemental File**

Oligonucleotide primers list

| **PRIMER** | **SEQUENCE** |
| --- | --- |
| ***miR-1249*** |  |
| miR-1249-M13-F | gtaaaacgacggccagCCAGGACTTTGGTGGATGTC |
| miR-1249-M13-R | caggaaacagctatgacGATCCACAGCCTGGGAAAT |
|  |  |
| **PRIMER** | **SEQUENCE** |
| ***IB2*** |  |
| IB2-M13-Ex 1-F | gtaaaacgacggccagGGCGTGTCACGAGGTGAG |
| IB2-M13-Ex 1-R | caggaaacagctatgacTGCGCAGGGAGCGGAGGGTCC |
| IB2-M13-Ex 2-F | gtaaaacgacggccagAGGGAGTCCAGGGTTGGT |
| IB2-M13-Ex 2-R | caggaaacagctatgacCCCTTGAGCTGGATCTGTGT |
| IB2-M13-Ex 3-F | gtaaaacgacggccagCCTGTAGACAGAGGGGCAGA |
| IB2-M13-Ex 3-R | caggaaacagctatgacTATGAACCCAGGCTCAGCTC |
| IB2-M13-Ex 4-F | gtaaaacgacggccagAGCTGAGCCTGGGTTCATAG |
| IB2-M13-Ex 4-R | caggaaacagctatgacATCCTCCCTGCCCTCCTC |
| IB2-M13-Ex 5-F | gtaaaacgacggccagGAGAGGGTGACCCCACCT |
| IB2-M13-Ex 5-R | caggaaacagctatgacGCAGGAGACAGGCACTGG |
| IB2-M13-Ex 5b-F-new | gtaaaacgacggccagCTGCTCAGCAACCTGGTG |
| IB2-M13-Ex 5b-R-new | caggaaacagctatgacCTGTCCTCTTCGCTGTCGT |
| IB2-M13-Ex 6-F | gtaaaacgacggccagAGTACACGCTGGTGGTGGAT |
| IB2-M13-Ex 6-R | caggaaacagctatgacGAGTCTGCACCTTCCTCTGG |
| IB2-M13-Ex 7-8-9-F | gtaaaacgacggccagAAGCACTACTCTGACTCTGTCTCC |
| IB2-M13-Ex 7-8-9-R | caggaaacagctatgacACCCTCAGCCTGCACTTG |
| IB2-M13-Ex 10-11-F | gtaaaacgacggccagCCTCTTCTCCCCCAACTTCT |
| IB2-M13-Ex 10-11-R | caggaaacagctatgacGGTTGGACTCTCAGGGTTTG |
| IB2-M13-Ex 11-F | gtaaaacgacggccagGCTAATGTCTGGGTGGAGGA |
| IB2-M13-Ex 11-R | caggaaacagctatgacTTTTGGTTTCAGCCCAAGC |
| IB2-M13-Ex 12-F | gtaaaacgacggccagCTCTGACGTGCCCTCCAC |
| IB2-M13-Ex 12-R | caggaaacagctatgacCCATTCTGCACCCACACC |
| IB2-M13-Ex 13-F | gtaaaacgacggccagCAGTTTGGGGGTTGAGGAC |
| IB2-M13-Ex 13-R | caggaaacagctatgacATGGTGGCCTCTTGAGACAT |
|  |  |
| **PRIMER** | **SEQUENCE** |
| ***RABL2B*** | (Exons 1 and 2 are not coding) |
| RABL2B-Ex 3-F | gtaaaacgacggccagCCTCTTGTTGATCTTCCCAGA |
| RABL2B-Ex 3-R | caggaaacagctatgacAAATGACCTGGACCCTCTCT |
| RABL2B-Ex 4-F | gtaaaacgacggccagGGTGAAAAAGTCTGGGACGA |
| RABL2B-Ex 4-R | caggaaacagctatgacAACAAGTGCGGAACAAAACC |
| RABL2B-Ex 5-F | gtaaaacgacggccagACACCCCTGCTCCCCACT |
| RABL2B-Ex 5-R | caggaaacagctatgacTTCTCCCTCTGGACTCTCTCC |
| RABL2B-Ex 6-F | gtaaaacgacggccagCACATGTGCTGTGGTTTGTG |
| RABL2B-Ex 6-R | caggaaacagctatgacACCCCTCAGGCCAGACTT |
| RABL2B-Ex 7-F | gtaaaacgacggccagGAGATGGGGTGCAGAAACAT |
| RABL2B-Ex 7-R | caggaaacagctatgacGGAAATGTATGAGGGCAGGT |
| RABL2B-Ex 8-F | gtaaaacgacggccagCCTTCTTCCCTTCCCTTGAC |
| RABL2B-Ex 8-R | caggaaacagctatgacACACCTGCAGTAAGCCCTTC |
| RABL2B-Ex 9-F | gtaaaacgacggccagGGACTGGGCAGAGTCCAAG |
| RABL2B-Ex 9-R | caggaaacagctatgacTTAGTGGGGCAAACTTAGGC |
| RABL2B-Ex 10-F | gtaaaacgacggccagTGTATCAGAGCTGCGGTTGA |
| RABL2B-Ex 10-R | caggaaacagctatgacCCATTCAGAGCTGGAGAGTTG |
|  |  |
| **PRIMER** | **SEQUENCE** |
| ***SHANK3*** |  |
| SHANK3-M13-1F | gtaaaacgacggccagGCGCTCCGTTCCCCGGCGCGA |
| SHANK3-M13-1R | caggaaacagctatgacCCTCCGCGAACCGCGGCCGAA |
| SHANK3-M13-2F | gtaaaacgacggccagGACCTGAGCTCACGAGCCCGCT |
| SHANK3-M13-2R | caggaaacagctatgacCTGCCGTGCCCTTCACTGGTC |
| SHANK3-M13-3F | gtaaaacgacggccagTCCACTGTGGTAGTATGACTG |
| SHANK3-M13-3R | caggaaacagctatgacTGGAACACATCACTGTACCAC |
| SHANK3-M13-4F | gtaaaacgacggccagGAGGAAGGCGGGTGATGTTCA |
| SHANK3-M13-4R | caggaaacagctatgacCCACCATTCTTCAGCACCTT |
| SHANK3-M13-5F | gtaaaacgacggccagTGTCTGTGAACCCAGAGTGC |
| SHANK3-M13-5R | caggaaacagctatgacGAGAAGCAGCTCACAGCAGA |
| SHANK3-M13-6F | gtaaaacgacggccagGGCTCTTGCCTGGTGATG |
| SHANK3-M13-6R | caggaaacagctatgacAGAGCCAGGACCCCCAAC |
| SHANK3-M13-7F | gtaaaacgacggccagGGGTCCTGGCTCTGTCTGTA |
| SHANK3-M13-7R | caggaaacagctatgacAGTATATCCACACTCGGTGCA |
| SHANK3-M13-8F | gtaaaacgacggccagGTGTGCATTCCTGTGTGCGCA |
| SHANK3-M13-8R | caggaaacagctatgacGGCTTCTGCACCCCTGCTGGT |
| SHANK3-M13-9F | gtaaaacgacggccagATCCCAGTTACAGACAAGAGT |
| SHANK3-M13-9R | caggaaacagctatgacCAATGTTCACTCAACACAGGC |
| SHANK3-M13-10+11F | gtaaaacgacggccagGCCTGGGCAAACTGGACAAGT |
| SHANK3-M13-10+11R | caggaaacagctatgacTCCCCAACCAGGAAGCCCTAG |
| SHANK3-M13-11altF | gtaaaacgacggccagCGCCCAGGAGCTGTATTC |
| SHANK3-M13-11altR | caggaaacagctatgacGGAGACCCCAGAGCCACT |
| SHANK3-M13-12F | gtaaaacgacggccagAGCTGGGAGAAAGTGGGAAGG |
| SHANK3-M13-12R | caggaaacagctatgacGTCACACACGTCCTATGTGTC |
| SHANK3-M13-13+14F | gtaaaacgacggccagACTGGTGACCAGCATGGGTGA |
| SHANK3-M13-13+14R | caggaaacagctatgacGGCAGAAGCAAGAAGCTGAAG |
| SHANK3-M13-15F | gtaaaacgacggccagACTCGGAGGTTGCTGTGTG |
| SHANK3-M13-15R | caggaaacagctatgacGTCCACCACAGGGCTCAG |
| SHANK3-M13-16F | gtaaaacgacggccagATCGAGGTGAGGTCGTTCTG |
| SHANK3-M13-16R | caggaaacagctatgacATGTGGGCCTGAAGTCAAAG |
| SHANK3-M13-17F | gtaaaacgacggccagACCTGAACAAGATCCTGGCAC |
| SHANK3-M13-17R | caggaaacagctatgacCACCCATTCACCTCTGACCTG |
| SHANK3-M13-18F | gtaaaacgacggccagGCATGTACCAACTGACTCCAG |
| SHANK3-M13-18R | caggaaacagctatgacCCATCACAGTCTCAGAGGGTC |
| SHANK3-M13-19F | gtaaaacgacggccagCTCTGTCAGCATCACGGGTG |
| SHANK3-M13-19R | caggaaacagctatgacCTCTCACCTCCGTCAAGAGG |
| SHANK3-M13-20F | gtaaaacgacggccagTCACCTCTGGCTTAGGAGGA |
| SHANK3-M13-20R | caggaaacagctatgacCTTCAAACCCAAGTCCACCCT |
| SHANK3-M13-21a1.1F 21a1aF | gtaaaacgacggccagAAGGCTGGCCTCTGTGGGAGG |
| SHANK3-M13-21a1.2R new 21a1aR | caggaaacagctatgacACCTGCAGCTGCTTCACC |
| SHANK3-M13-21a1.2F new 21a1bF | gtaaaacgacggccagGCGCCCTACTACTTCGACTC |
| SHANK3-M13-21a1.3R 21a1bR | caggaaacagctatgacACGGACAGGAACACAGTGG |
| SHANK3-M13-21a2F | gtaaaacgacggccagCAAGAGCCCCCTGGTGAAGCA |
| SHANK3-M13-21a2R | caggaaacagctatgacGCTCTCGGGCAGCCAGGGCAA |
| SHANK3-M13-21a3F | gtaaaacgacggccagTGCCCTGAAGCCGTTGGTCAG |
| SHANK3-M13-21a3R | caggaaacagctatgacACCTTCTCTGCCTCCCTGCGA |
| SHANK3-M13-21a4F | gtaaaacgacggccagCCTGTTTGTGGATGTACAGGCC |
| SHANK3-M13-21a4R | caggaaacagctatgacGACGCTGAGGATCATGGACT |
| SHANK3-M13-21a5F | gtaaaacgacggccagTCAGCGTCCTGGACACATC |
| SHANK3-M13-21a5R | caggaaacagctatgacCTGGCTCTTCCTCTGAGCTG |
| SHANK3-M13-21a6F | gtaaaacgacggccagATGCAGTCAGCGGCTGTG |
| SHANK3-M13-21a6R | caggaaacagctatgacCTCCTCCCTGGTCTCCTCAT |
| SHANK3-M13-21a7F new | gtaaaacgacggccagGAGCTGGTGTTTGCTGTGAA |
| SHANK3-M13-21a7R new | caggaaacagctatgacCCTCCTCCACCCCAGAGT |
| SHANK3-M13-21a8F | gtaaaacgacggccagCTCAGGGAAGCCCAGCAGTGA |
| SHANK3-M13-21a8R | caggaaacagctatgacAGTTGGTTTGTCGTCTTCAGG |
| SHANK3-M13-21a9F | gtaaaacgacggccagGCCTGAAGACGACAAACCAA |
| SHANK3-M13-21a9R | caggaaacagctatgacAGAACAGACAAGAGGAATGAC |
| SHANK3-M13-21b1F | gtaaaacgacggccagGAGGAGCCCTTCGGGCCCGTG |
| SHANK3-M13-21b1R | caggaaacagctatgacACTCTGTCCTCCAAGGCTGA |
| SHANK3-M13-21b2F | gtaaaacgacggccagGTCAGCCTTGGAGGACAGAG |
| SHANK3-M13-21b2R | caggaaacagctatgacCTACGGGCACCTTCAACACT |
| SHANK3-M13-21cF | gtaaaacgacggccagGTCTCGAAGGGAAACATGAAC |
| SHANK3-M13-21cR | caggaaacagctatgacCAGTGTCCATGTCTGACTTCC |
| SHANK3-M13-22a1F | gtaaaacgacggccagCCGTAGGATCCCACCCTTTA |
| SHANK3-M13-22a1R | caggaaacagctatgacGGCCTCACCGAGTACGAG |
| SHANK3-M13-22a2F | gtaaaacgacggccagGTACTCGGTGAGGCCCAGT |
| SHANK3-M13-22a2R | caggaaacagctatgacGAGAGGCTGGACGACTTGAG |
| SHANK3-M13-22a3F | gtaaaacgacggccagACCATCCTCAAGTCGTCCAG |
| SHANK3-M13-22a3R | caggaaacagctatgacCCTAGGTGGATGCTCTCCAG |
| SHANK3-M13-22a4F | gtaaaacgacggccagGCTGGAGAGCATCCACCT |
| SHANK3-M13-22a4R | caggaaacagctatgacCGATTCATGCAACATTCCTG |

Bioinformatic websites

| **NAME** | **WEB ADDRESS** |
| --- | --- |
| Mutation Taster | <http://www.mutationtaster.org/> |
| MICO | <http://mico.ggc.org/MICO/> |
| Splice Site Calculator | http://192.168.50.223/splicing/maxent.cgi |
| Splice Site Score Calculation | <http://rulai.cshl.edu/new_alt_exon_db2/HTML/score.html> |
| RESCUE-ESE | <http://genes.mit.edu/burgelab/rescue-ese/> |
| Combined Annotation Dependent Depletion (CADD) | <http://cadd.gs.washington.edu/home> |
| 1000genomes | <http://www.1000genomes.org/> |
| NHLBI Exome Variant Server | <http://evs.gs.washington.edu/EVS/> |
